# Dynamic Screw Lead Accuracy Measurement: Research on the Compensation of the Workpiece Placed Away from the Ideal Position

**DOI:** 10.3390/s24216829

**Published:** 2024-10-24

**Authors:** Lin Tao, Lin Zhang, Xin Wang, Xianguang Fan, Hua Shi

**Affiliations:** 1 School of Opto-Electronic and Communication Engineering, Xiamen University of Technology, Xiamen 361005, China; taolin@xmut.edu.cn (L.T.); zhanglin603@aliyun.com (L.Z.); 2 School of Aerospace Engineering, Xiamen University, Xiamen 361005, China; xinwang@xmu.edu.cn (X.W.); fanxg@xmu.edu.cn (X.F.)

**Keywords:** screw, dynamic accuracy measurement, eccentricity, placing tilt, bias errors compensation

## Abstract

The screw, a critical element in a variety of transmission mechanisms, significantly influences the performance of the transmission process. Accurate measurement of screw lead is crucial for ensuring the quality of transmission equipment. However, the measurement process can be affected by the precision limits of the measuring instruments and the challenges of manual fine adjustments. This can lead to the screw being misplaced, introducing errors due to the off-center positioning of the workpiece. Such errors can hinder the achievement of high-precision measurements. This research aims to reduce the time needed to adjust for workpiece misalignment and to improve the accuracy of screw lead measurement through error compensation. This study starts by examining two specific scenarios that can cause workpiece misalignment in screw lead accuracy measurements: the tilting of the workpiece and the misalignment of the workpiece axis relative to the circular grating axis. Then, a mathematical model to quantify this misalignment and measure the associated parameters is developed. Based on the measured parameters, a computational model is established to compensate for the bias error under these conditions. This method allows for efficient and precise measurement of the screw lead even when the workpiece is not perfectly aligned. Calibrated screws and a digital micrometer are used to conduct experiments on workpiece misalignment. By comparing measurements with and without error compensation, the effectiveness of the compensation method in enhancing measurement accuracy is demonstrated.

## 1. Introduction

Screws are efficient transmission devices that facilitate the conversion between linear and rotational motion. They are extensively utilized in key operational components of equipment such as automobiles, industrial robots, computer numerical control (CNC) machines, and precision instruments [1,2]. As precision manufacturing and industrial technology progresses, the pursuit of enhanced transmission accuracy for screws has become increasingly significant. To achieve this, it is not only imperative to refine the manufacturing precision of screws but also to ensure that the methods used for assessing their accuracy are equally rigorous. Lead accuracy is one of the most significant parameters for evaluating the performance of a screw assembly [3,4].

The lead error of a screw is defined as the discrepancy between the screw’s actual lead and its theoretical lead during the transmission process. This deviation is primarily due to manufacturing tolerances, installation errors, and wear accrued over operational use [5,6,7]. At present, dynamic measurement methods for screw lead have emerged as the standard approach within the field, reflecting the broader trend towards measurement equipment that is more precise, versatile, and automated [8,9,10]. In an ideal scenario, existing measurement systems would require the workpiece to be positioned perfectly for accurate readings. However, the practical manufacturing tolerances of these instruments, coupled with the limitations of manual fine-tuning, often lead to the workpiece being misaligned from this ideal position. Such misalignment complicates the task of achieving optimal workpiece installation and, as a result, impedes the efficiency of precision measurement for screws. Therefore, there is a clear need for research that addresses the reduction in errors caused by fixture misalignment, even when the precision requirements for workpiece clamping are relaxed. The goal of this research is to improve both the accuracy and the efficiency of screw lead measurements, despite the challenges posed by less-than-ideal workpiece positioning.

Early researchers dedicated significant efforts to reducing positional bias errors in the manufacturing process. Yang et al. [11] analyzed bias in screw machining centers, spindle runout, and the contact conditions of incompatible center holes. They developed an automatic, adjustable center for two-dimensional positioning to mitigate alignment errors during the machining of workpieces supported by two centers. Zhang et al. [12] developed predictive models for the positioning errors of screw transmission systems, accounting for manufacturing and installation variables. Using multivariate linear regression to ascertain the model coefficients, they successfully improved the system’s positioning precision by compensating for errors on the X-axis of a three-axis CNC mill. Li et al. [13] introduced a comprehensive compensation strategy for dynamic positioning errors in machining processes, focusing on the rectification of biases inherent in manufacturing. Concurrently, Pai et al. [14] executed a simulation-based analysis to assess the bias precision, linearity, and stability of screw measurement systems. They further employed Monte Carlo simulations to construct linear variance and probability density functions for deviations, providing a robust framework for evaluating the stability and reliability of the measurement apparatus. Wang et al. [15] devised a method for detecting bias errors in screws by monitoring the average vibration amplitude induced by the rotational frequency of the screw, thereby enhancing the alignment precision of machine tools and automated equipment. Feng et al. [16] utilized a micrometer to measure the Y-axis deviation caused by bias along the feed direction (X-axis). For each preload and bias setting, vibration signals are ascertained from the installed MEMS vibration detection module while the controller actuates the worktable. By analyzing the vibration signals of the detection module mounted on the linear guide block, it has been observed that characteristic frequencies within specific frequency bands can serve as indicators of displacement. Han et al. [17] studied the calculation method of clamping eccentricity parameters when large-sized workpieces were installed with eccentricity and proposed an error compensation method based on this. Wang et al. [18] examined the error model induced by tilted installations and developed an error compensation strategy. Their research substantiated that the compensation technique significantly improves the precision of workpiece measurements.

While previous research has shown significant success in compensating for bias errors, it has also revealed certain inherent limitations. Mainly, the current research has largely focused on compensating for eccentricity errors in the context of workpiece installation bias. Moreover, the determination of bias magnitudes has been carried out manually, which significantly limits the efficiency of workpiece measurement procedures. Building on the aforementioned research, two specific models of workpiece placement bias are examined within screw lead accuracy measurement systems: workpiece tilting and the misalignment of the workpiece axis from the central axis of the circular grating. Within the coordinate system of the measuring instrument, geometric models are developed to determine the tilt angle and the degree of eccentricity. These models form the foundation for subsequent analysis and the development of an error compensation strategy. Then, measurement models are compared under ideal conditions with those under biased clamping conditions, highlighting similarities and differences. This comparison leads to the development of a measurement model for screw lead accuracy that incorporates installation bias. The model facilitates achieving high-precision measurement results even with clamping bias, thus enhancing both the accuracy and efficiency of the measurement process.

## 2. Materials and Methods

### 2.1. Principles of Dynamic Measurement

Dynamic measurement systems are used for assessing the lead error of the screw under test, which is ascertained by juxtaposing its actual helical trajectory against a predefined standard helix. In accordance with the measurement principle outlined, the error of the screw can be determined by the following equation:

(1) $$∆=x-\frac{\theta }{2\pi }T$$

where ∆ is defined as the screw error, *x* denotes the displacement of the measuring probe along the screw’s axial path, T is the theoretical lead, and θ is the angular rotation of the screw.

The lead accuracy grade of the screw is evaluated by the error ∆ of Equation (1) based on the standards defined by the International Organization for Standardization (ISO), which is commonly used as a reference standard denoted as GB in China [6,8].

**Figure 1:**
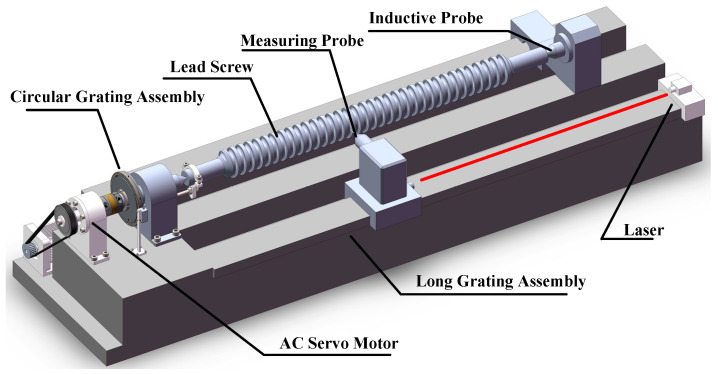
Screw lead accuracy measurement system.

As depicted in Figure 1, the screw accuracy measurement system employs a circular grating to measure angular displacement and a combination of a long grating and inductive sensors to measure the moved distance. A highly stable AC servo motor is utilized within the transmission component to achieve smooth control over the screw’s rotational speed through frequency conversion. The circular grating synchronizes with the rotation of the screw under test to measure its angular rotation, while a laser interferometer probe, fixed on a nut carriage, measures the linear displacement of the carriage as it moves along with the nut. This setup allows for the precise measurement of both rotational and translational movements, which are critical for assessing the screw’s lead accuracy.

**Figure 2:**
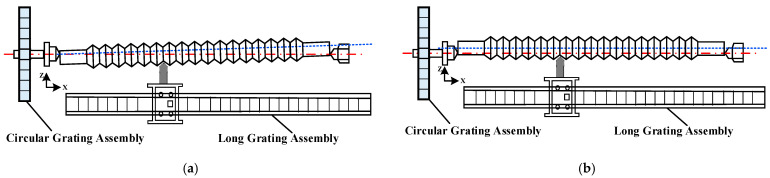
Schematic diagram of screw placement deviating from ideal position. (**a**) Inclined placement of screw; (**b**) installation eccentricity of circular grating. The red dashed line is the rotation axis during measurement, and the blue dashed line is the center axis of the screw.

Due to the manufacturing accuracy of the screw lead measurement system itself and the limitation of manual micro feed capacity during workpiece placement, the placement of the lead during measurement deviates from the ideal position. There are usually two situations, as shown in Figure 2. Figure 2a shows the inclination of the workpiece placement, that is, the workpiece axis forms a certain angle with the circular grating axis, and Figure 2b shows the deviation of the workpiece axis from the circular grating axis.

### 2.2. Compensation for Screw Installation Tilt

#### 2.2.1. Geometric Model for Screw Installation Tilt

**Figure 3:**
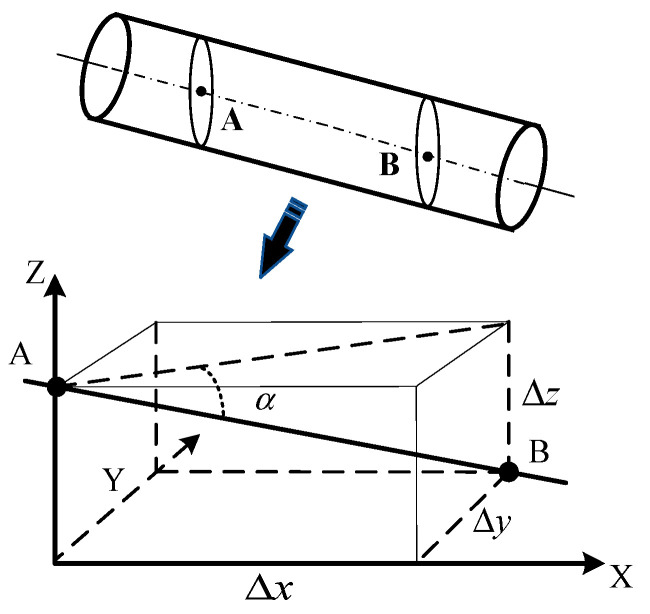
Geometric modeling of workpiece tilt angle. Point A and point B are two points on the axis of the workpiece.

If the line connecting the centers of dead centers and live centers is not parallel to the direction of the standard measurement, angular positioning errors will occur [18]. The geometric modeling of axis tilt is shown in Figure 3, where A and B are two points on the central axis of the workpiece, and α is the angle between the central axis and the ideal axis, also known as the tilt angle of the screw axis.

Make the coordinates of point A in the diagram $\left(x_{1},y_{1},z_{1}\right)$, and point B $\left(x_{2},y_{2},z_{2}\right)$. The tilt angle α of the screw’s central axis is

(2) $$\alpha =\tan^{-1}\frac{∆z}{\sqrt{{∆x}^{2}+{∆y}^{2}}}$$

where $∆x=x_{1}-x_{2}$, $∆y=y_{1}-y_{2}$, and $∆z=z_{1}-z_{2}$.

#### 2.2.2. Computational Approach for Acquiring the Tilt Angle

**Figure 4:**
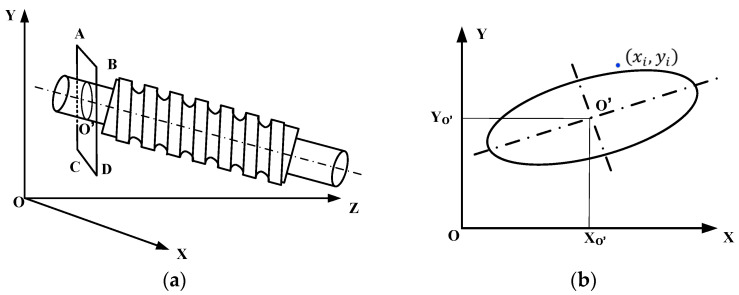
Schematic diagram of workpiece placement tilting. (**a**) Three-dimensional workpiece inclination; (**b**) two-dimensional planar ellipse. ABCD is a plane parallel to XOY, and $O^{\prime }$ is the intersection point between the plane ABCD and the workpiece axis.

When a cylinder is tilted, any plane cutting through it will intersect the surface of the tilted cylinder to form a curve [19]. The projection of the curve onto the XOY plane, as shown in Figure 4, will appear elliptical in shape. The center of this ellipse, denoted as $O^{\prime }$, corresponds to the intersection point of the plane with the central axis of the cylinder.

In the two-dimensional plane coordinate system depicted in Figure 4b, the general form of an ellipse can be represented by the following curve equation:

(3) $$Ax^{2}+Bxy+Cy^{2}+Dx+Ey+F=0$$

where A, B, C, D*,* E*,* and F are constants, and A and B cannot both be 0 at the same time.

The least squares technique primarily involves identifying a set of parameters that minimize the distance metric between data points and an elliptical curve. The widely used algebraic distance of a point $\left(x_{i},y_{i}\right)$ within a plane to a curve represented by the equation $f\left(x,y\right)=0$ is given by the value of $f\left(x_{i},y_{i}\right)$, which is expressed as

(4) $$f\left(x_{i},y_{i}\right)=A{x_{i}}^{2}+Bx_{i}y_{i}+C{y_{i}}^{2}+Dx_{i}+Ey_{i}+F$$

Assume N points on the surface of the workpiece parallel to the ABCD plane are collected, denoted as ${(x}_{i},y_{i}),i=1,\cdots N$. For the general equation of an ellipse presented in Equation (3), the extraction of the respective coefficients is facilitated through the least squares method. Specifically, the determination of these coefficients is achieved by minimizing the objective function as delineated in Equations (5) and (6).

(5) $$Minimize:\;\sum_{i=i}^{N}{f(x_{i}{,y}_{i})}^{2}$$

(6) $$\underset{A,B,C,D,E,F}{\min}\sum_{i=i}^{N}{\left(Ax_{i}^{2}+Bx_{i}y_{i}+Cy_{i}^{2}+Dx_{i}+Ey_{i}+F\right)}^{2}$$

In accordance with the principle of extremization, to minimize the value of f(A,B,C,D,E), it is required that

(7) $$\frac{\partial f}{\partial A}=\frac{\partial f}{\partial B}=\frac{\partial f}{\partial C}=\frac{\partial f}{\partial D}=\frac{\partial f}{\partial E}=0$$

Consequently, this results in a system of linear equations. Utilizing an algorithmic approach tailored for the resolution of such systems, specifically the full-pivoting Gaussian elimination method, while also integrating the pertinent constraint conditions, enables the precise determination of the coefficients within the equation. This methodology aligns with established practices in numerical analysis for parameter estimation within a predefined model, under a set of specified constraints.

After establishing the general equation of the ellipse, the coordinates of the ellipse’s centroid can be readily computed. This information facilitates the determination of point coordinates along the central axis of the screw. As depicted in Figure 4b, which illustrates the general form of a two-dimensional planar ellipse, the coordinates of the ellipse’s center $O^{\prime }$, denoted as $\left(x_{O^{\prime }},y_{O^{\prime }}\right)$, can be derived from the general equation of the ellipse.

(8) $$\left\{\begin{array}{c} x_{O^{\prime }}=\frac{BE-2CD}{4AC-B^{2}}\\ y_{O^{\prime }}=\frac{BD-2AE}{4AC-B^{2}} \end{array}\right.$$

#### 2.2.3. Acquisition of the Tilt Angle and Error Compensation

**Figure 5:**
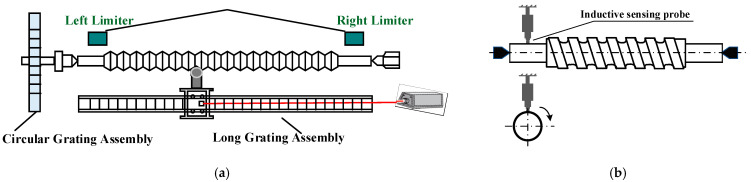
Installation schematic diagram of inductive sensor for axial tilt measurement. (**a**) Installation position of inductance probe; (**b**) installation method of inductance probe.

An inductive sensing probe is utilized, installed at the left and right limiters of the measurement system as shown in Figure 5. Upon activation, the system completes a full rotation, acquiring data points at every 60-degree interval.

The surface points collected from the left limiter position are used to ascertain the centroids of elliptical cross-sections by employing the elliptical fitting technique, as described at point A in Figure 3. Meanwhile, the same method is used to obtain point B as described in Figure 3 on the right limiter. This analytical approach subsequently facilitates the computation of the axis’s inclination, i.e., α, by Equation (2).

**Figure 6:**
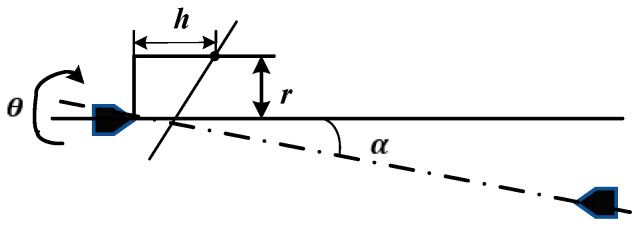
Schematic diagram of calculation of the angle positioning error.

The schematic diagram of the parameters corresponding to the calculation of tilt angle compensation is shown in Figure 6. Let h denote the displacement of the measuring probe along the screw’s axial path and r denote the measured radius of rotation.

When the workpiece tilts, there will be angular positioning errors, which were first mentioned in the manufacturing accuracy error compensation based on references [10,16,18]. At this point, it is used to compensate for the angle deviation, where the angle positioning error can be obtained as follows:

(9) $$∆\theta_{1}=\frac{h\alpha }{r}\sin\theta +(\frac{1}{4}+\frac{h^{2}}{2r^{2}})\alpha^{2}\sin\left(2\theta \right)$$

Considering the deviation in rotation angle caused by the inclination of the test screw, combined with Equation (1) for the lead accuracy error of the screw, the measurement model after error compensation can be obtained as follows:

(10) $${∆}_{1}=x-\frac{\theta +∆\theta_{1}}{2\pi }T$$

where ${∆}_{1}$ is defined as the screw error after tilt compensation, x denotes the distance along the screw’s axial path, T is defined as the theoretical lead, θ is the angle measured by a circular grating, and $∆\theta_{1}$ is the angle positioning error caused by tilt.

### 2.3. Compensation for Eccentricity in Screw Installation

**Figure 7:**
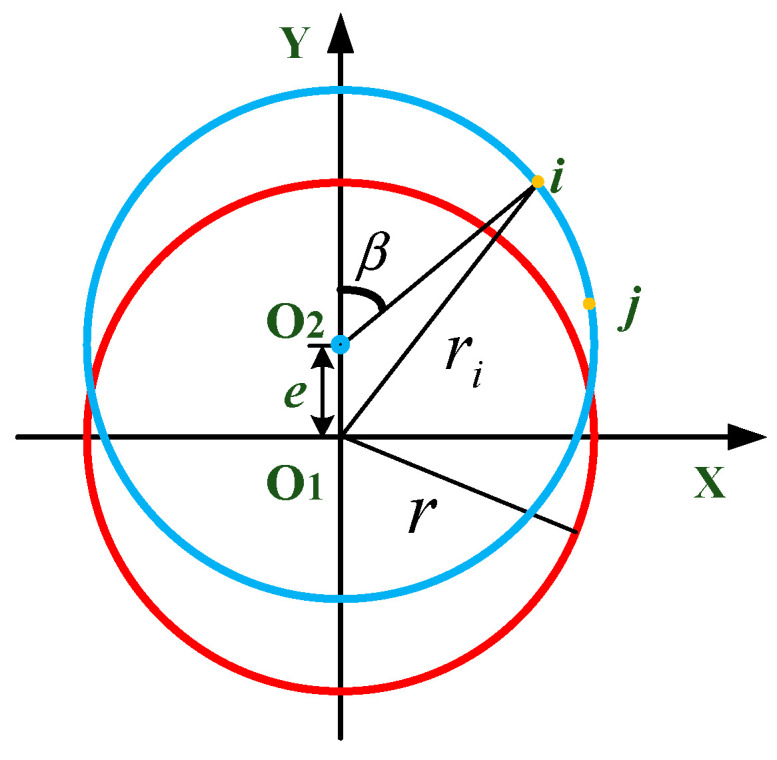
Schematic diagram of installation eccentricity of circular grating. The red circle represents the ideal position where the workpiece is installed, and the blue circle represents the actual position where the workpiece is installed eccentrically.

When a circular grating serves as the sensing element, the presence of installation eccentricity leads to a bias between the geometric center of the grating disk and the axis of rotation being measured. This bias can significantly impair the precision of the measurement [18]. Let $O_{1}$ denote the geometric center of the grating disk and $O_{2}$ denote the axis of rotation. Figure 7 depicts the installed state of the grating disk, highlighting an eccentricity e, which pertains to its rotational bias.

When $O_{1}$ and $O_{2}$ coincide, the rotation radius of the system is equal to the geometric radius of the grating disk. However, due to the deviation of the workpiece placement from the center of the circular grating, the actual measured radius of rotation on point i deviates. The calculation of the rotational radius $r_{i}$ can be derived by the sine rule referring to references [6,10,17], which describe the same situation, and can be expressed as

(11) $$r_{i}=r\cdot \cos\sin^{-1}(\frac{e}{r}\sin\beta )-e\cdot \cos\beta$$

where r is defined as the geometric radius of the grating disk and e and β are defined as the eccentricity of the measured axis of rotation relative to the geometric center of the grating disk.

As e≪r and $\cos\sin^{-1}(\frac{e}{r}\sin\beta )\approx 1$,Equation (10) can be simplified to

(12) $$r_{i}=r-e\cdot \cos\beta$$

Then, when $O_{2}$ is considered as the rotation center, the arc length traversed by the grating disk relative to the measuring head as it rotates past point γ can be calculated by the following equation:

(13) $$∆x_{1}=\int_{\beta_{1}}^{\beta_{2}}r_{i}d\beta =\int_{\beta_{1}}^{\beta_{2}}(r-e\cdot \cos\beta )d\beta ={\left(r\cdot \beta -e\cdot \sin\beta )\right|}_{\beta_{1}}^{\beta_{2}}$$

When $O_{1}$ is considered as the rotation center, the arc length traversed by the grating disk relative to the measuring head as it rotates past point γ can be calculated by the following equation:

(14) $$∆x_{2}=\int_{\beta_{1}}^{\beta_{2}}rd\beta ={r\cdot \beta |}_{\beta_{1}}^{\beta_{2}}$$

Then, the displacement deviation is

(15) $${∆e=∆x_{1}-∆x_{2}=(r\cdot \beta -e\cdot \sin\beta )|}_{\beta_{1}}^{\beta_{2}}-{r\cdot \beta |}_{\beta_{1}}^{\beta_{2}}={-e\cdot \sin\beta |}_{\beta_{1}}^{\beta_{2}}$$

The error, when expressed in radians, is as follows:

(16) $$∆\theta =\frac{e\cdot \sin\beta }{r_{i}}=\frac{e\cdot \sin\beta }{r-e\cdot \cos\beta }$$

As e≪r, Equation (16) for describing rotation angle positioning error can be simplified to

(17) $${∆\theta }_{2}=\frac{e\cdot \sin\beta }{r}$$

Considering the deviation in rotation angle caused by eccentricity, combined with Equation (1) for the lead accuracy error of the screw, the measurement model after error compensation can be obtained as follows:

(18) $${∆}_{2}=x-\frac{\theta +∆\theta_{2}}{2\pi }T$$

where ${∆}_{2}$ is defined as the screw error after eccentricity compensation, x denotes the distance along the screw’s axial path, T is defined as the theoretical lead, θ denotes the angle measured by a circular grating, and $∆\theta_{2}$ is the angle positioning error caused by eccentricity.

## 3. Experiment Results

**Figure 8:**
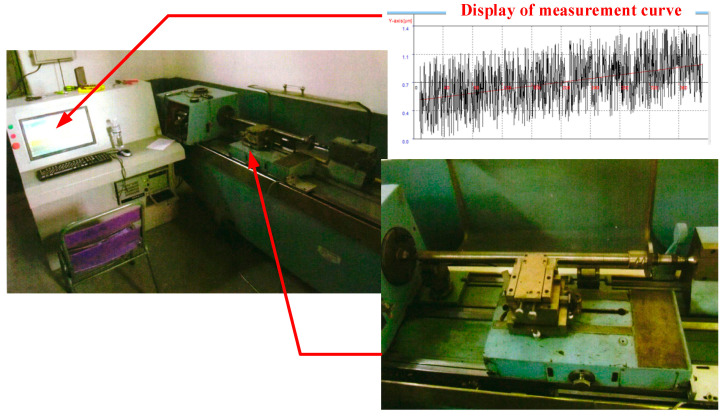
Interface of screw lead accuracy measurement system.

The lead accuracy measurement system used in the experiment is displayed in Figure 8. The experimental acceptance inspection and calibration are based on the latest revised standards GB/T17587.3: 2017 [20] and JB 2886-2008-T [21] and comply with international inspection standards.

The least squares method should be used to calculate the regression coefficients for the regression line of the travel error curve and evaluate the accuracy level using the travel accuracy (2π travel variation), V300 (travel variation within any travel distance of 300), and Vu (average travel deviation within effective travel). The calibrated right-handed C5-level P-type ball screw (marked as PBS-C5) and 7-level trapezoidal screw (marked as TS-7) are employed for lead accuracy measurement. The corresponding accuracy indicators are shown in Table 1. Both screws have a lead of 500 mm and a nominal lead of 4 mm. The distance between the left and right limit switches is set at 560 mm. The comparative assessment method involves the deactivation and activation of error compensation.

A. Experiment for screw installation tilt

Before the measurement process begins, it is imperative to initially define the instrument coordinate system at the center of the screw measurement setup. The workpiece is then carefully positioned on the measurement platform and adjusted to the designated location with the aid of a digital micrometer. With the screw correctly positioned, the system transitions to the measurement interface to start data acquisition. To substantiate the effectiveness of the compensation for workpiece bias errors, the right end of the workpiece is adjusted to achieve a predetermined angular tilt. To measure the bias angle, the inductive probes at both end limiters are pressed against the non-tooth face of the screw. As the grating sensor completes one rotation (2π), coordinate data from six points on the cylindrical surface are collected. These data are then used to calculate the bias angle of the screw’s geometric center axis. Subsequently, with this bias angle determined, the collected data are processed through the program, both with error compensation disabled and enabled, to evaluate the effectiveness of the compensation mechanism. To mitigate the impact of random errors, this study conducts repeated tests at the same location 10 times and adopts the mean value. The measurement results are presented in Table 2 and Table 3. The angle value is calculated to three decimal places.

B. Experiment for eccentricity of screw installation

To demonstrate the efficacy of eccentricity error compensation for the circular grating, this study readjusts the position of the screw, monitoring the position of the screw’s geometric center axis with a digital micrometer. The screw’s geometric center axis is deviated from the main axis of the circular grating by a specific dimension, and the lead accuracy at this position is measured repeatedly. Additionally, to elucidate the impact of the degree of eccentricity on workpieces of different diameters, two7-level trapezoidal screws are selected, with diameters of 5 cm and 8 cm, respectively, for measurement, denoted as TS-7-5cm and TS-7-8cm. To reduce the influence of random errors, this study repeats the test at the same position ten times and takes the average value as the result. The measurement results are presented in Table 4 and Table 5. In the measurement, it is considered acceptable if the screw’s geometric center axis is within 10 µm of the main axis of the screw measurement center.

## 4. Discussion

A. Discussion for the experiment of screw installation tilt

**Figure 9:**
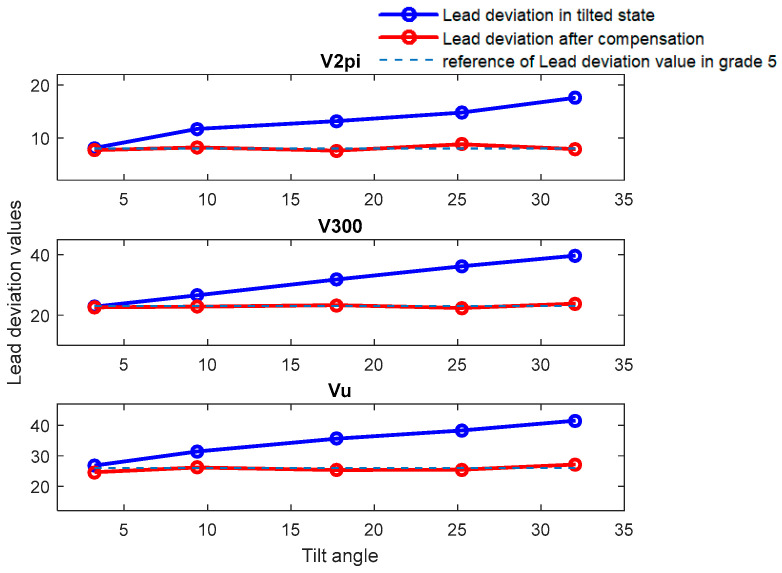
Comparative diagram of deviation before and after compensation at various tilt angles for PBS-C5.

**Figure 10:**
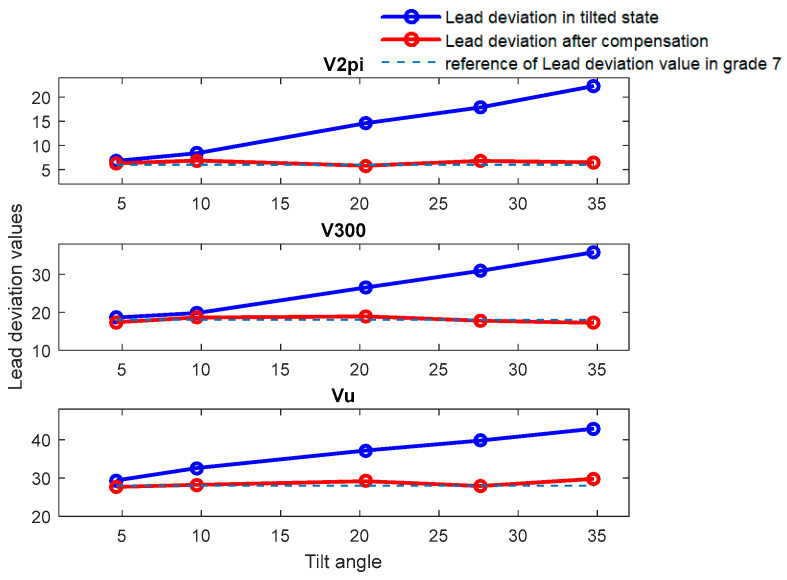
Comparative diagram of deviation before and after compensation at various tilt angles for TS-7.

Figure 9 and Figure 10 illustrate comparative diagrams of the lead accuracy measurement indicators before and after compensation for PBS-C5 and TS-7, respectively. The corresponding accuracy indicators are referred to in Table 1.

It can be observed from Figure 9 that when measuring PBS-C5, the precision grade evaluation is 5, which is consistent with the reference at a tilt angle of 3.25°. However, when the tilt angle exceeds 9.32°, the precision grade evaluation without compensation, with the lowest rating exceeding grade 5, significantly deviates from the actual precision grade of the workpiece. Concurrently, the higher the degree of eccentricity, the lower the assessed precision grade. Then, after applying error compensation, the error is reduced and the level of error accuracy evaluation reaches level 5, which is consistent with the calibration level.

Similar observations are made from Figure 9 when measuring TS-7, where the precision grade evaluation of lead accuracy without error compensation, with the lowest rating being grade 8 when the angle is higher than 9.72°, again significantly deviates from the actual precision grade of the workpiece. After compensation, the values approach the calibrated grade 7 precision level. Meanwhile, the precision grade evaluation of both screws shows a phenomenon where the higher the degree of tilt angle, the greater the error, and the lower the assessed precision grade. In addition, the precision grade evaluation meets the calibration value when the tilt angle is less than 4.64°.

B. Discussion for the experiment of eccentricity of screw installation

**Figure 11:**
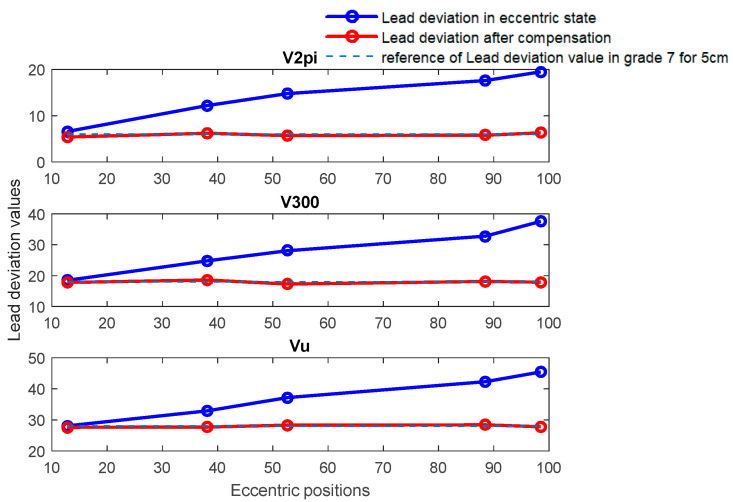
Comparative diagram of deviation before and after compensation at various eccentric positions for TS-7-5cm.

**Figure 12:**
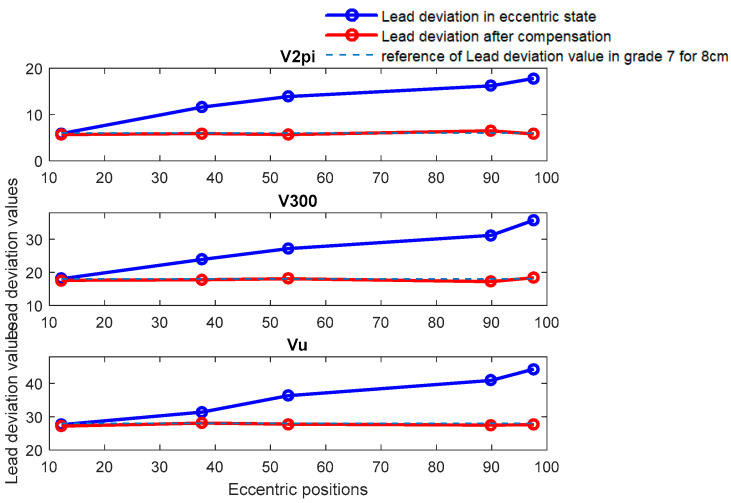
Comparative diagram of deviation before and after compensation at various eccentric positions for TS-7-8cm.

The experiment utilized two calibrated grade 7 screws, with diameters of 5 cm and 8 cm, respectively, to complete the lead accuracy measurement. Figure 11 and Figure 12 show comparative diagrams of lead accuracy measurement indicators before and after compensation in an eccentric state for TS-7-5cm and TS-7-8cm, respectively. Figure 11 illustrates the comparison of lead accuracy errors before and after eccentricity compensation. It can be observed that without compensation for eccentricity error, the precision grade is estimated to be 8 when the eccentricity position is 12.8 μm and the deviation angle is 337.07°, which is inconsistent with the actual calibration level. At the same time, the higher the degree of eccentricity, the worse the accuracy level evaluation, and the greater the deviation from the actual calibration situation. Then, after applying error compensation, the error is reduced and the level of error accuracy evaluation reaches level 7, which is consistent with the calibration level. The same phenomenon can also be observed in Figure 12 as in Figure 11.

Meanwhile, it can be observed that the error caused by installation eccentricity is inversely proportional to the radius R of the circular grating. Therefore, for the same installation eccentricity, a larger radius of the disk results in a lesser impact on measurement accuracy.

## 5. Conclusions

To boost the accuracy and efficiency of dynamic screw lead measurement systems and minimize the time spent on adjusting fixture biases, two specific bias models are explored: the tilt of the screw and the misalignment of its axis from the circular grating’s center. Leveraging the geometric properties of these biases, a method to calculate bias parameters by measuring the coordinates of points along the calibrated workpiece’s central axis is established. This approach allows us to derive a formula for compensating bias errors and to establish a measurement model for screw lead accuracy under biased fixture conditions. Using a calibrated workpiece and a digital micrometer, we conducted experiments to assess lead accuracy under various tilt and eccentricity scenarios. The findings show that when the tilt angle exceeds 9.37°, the accuracy, with post-bias compensation, matches the calibrated standards. Likewise, for misalignments greater than 12.4 µm, the compensated accuracy aligns with the calibration grade. These results confirm that even with reduced fixture precision, our system can determine bias parameters from the mathematical model and compensate for errors, thus providing a theoretical basis for achieving high-precision, efficient dynamic screw lead measurements.

## Figures and Tables

**Table 1:** Reference for calibrating workpiece indicators (μm).

Screws	$V_{2\pi }$	$V_{300}$	$V_{u}$
PBS-C5	8	23	26
TS-7	6	18	28

**Table 2:** Results of lead accuracy measurement for PBS-C5 before and after compensation (μm).

Bias Angle	$V_{2\pi }$		$V_{300}$		$V_{u}$	
	Before	After	Before	After	Before	After
3.25°	8.1	7.7	22.8	22.6	26.8	24.6
9.32°	11.7	7.2	26.6	21.8	31.4	26.1
17.75°	13.2	6.6	31.8	22.3	35.6	25.3
25.35°	14.8	7.8	36.2	22.7	38.3	25.4
31.72°	17.6	6.9	39.7	23.8	41.5	27.1

**Table 3:** Results of lead accuracy measurement for TS-7 before and after compensation (μm).

Bias Angle	$V_{2\pi }$		$V_{300}$		$V_{u}$	
	Before	After	Before	After	Before	After
4.64°	6.8	6.3	18.6	17.4	29.4	27.7
9.72°	8.4	6.9	19.8	18.6	32.6	28.2
20.36°	14.6	5.8	26.5	18.9	37.2	29.2
27.65°	17.9	6.8	30.9	17.8	39.8	27.9
34.72°	22.3	6.5	35.8	17.2	42.9	29.8

**Table 4:** Lead accuracy measurement results for TS-7-5cm at various eccentric positions (μm).

Eccentric Positions		$V_{2\pi }$		$V_{300}$		$V_{u}$	
*e* (μm)	β	Before	After	Before	After	Before	After
12.8	337.07°	8.1	7.7	22.8	22.6	26.8	24.6
38.1	175.65°	11.7	7.2	26.6	21.8	31.4	26.1
52.7	333.92°	13.2	6.6	31.8	22.3	35.6	25.3
88.5	287.73°	14.8	7.8	36.2	22.7	38.3	25.4
98.5	129.95°	17.6	6.9	39.7	23.8	41.5	27.1

**Table 5:** Lead accuracy measurement results for TS-7-8cm at various eccentric positions (μm).

Eccentric Positions		$V_{2\pi }$		$V_{300}$		$V_{u}$	
*e* (μm)	β	Before	After	Before	After	Before	After
12.2	337.07°	5.9	5.7	18.1	17.6	27.6	27.2
37.6	175.65°	11.6	5.9	23.9	17.8	31.4	28.1
53.2	333.92°	13.9	5.7	27.2	18.1	36.3	27.8
89.9	287.73°	16.2	6.5	31.2	17.2	40.9	27.5
97.6	129.95°	17.8	5.8	35.8	18.4	44.3	27.6

## Data Availability

The original contributions presented in the study are included in the article, further inquiries can be directed to the corresponding author.
